# Health of Louisville

**Published:** 1852-02

**Authors:** 


### HEALTH OF LOUISVILLE.

A year ago cholera was succeeded in this city by small-pox, which prevailed for a time to an extent probably never before known in Louisville. The exanthemata this season have been of a different character. We have heard of a good many cases of scarlatina, and measles is now epidemic, but scarcely a case of small-pox has been reported. For the most part scarlet fever has been mild, but cases have occurred of the worst type. We saw a little boy dying one evening at 7 o'clock, who had been attacked the previous day about noon. Suppuration of the parotid glands was an attendant upon some of the cases. The intense cold of the two past months has had its effect in the multiplication of cases of pneumonia and similar affections, but, except measles, the city, we believe, is clear of all epidemic complaints.

